# Lymph nodes as gatekeepers of autoimmune diseases

**DOI:** 10.1136/rmdopen-2024-004097

**Published:** 2024-12-10

**Authors:** Aoife M O'Byrne, Lisa G M van Baarsen

**Affiliations:** 1 Rheumatology & Clinical Immunology, Amsterdam UMC, University of Amsterdam, Amsterdam, The Netherlands; 2 Experimental Immunology, Amsterdam UMC, University of Amsterdam, Amsterdam, The Netherlands; 3 Amsterdam Institute for Immunology and Infectious diseases, Amsterdam, The Netherlands; 4 Amsterdam Rheumatology and Immunology Centre (ARC), Amsterdam, The Netherlands

**Keywords:** Autoimmunity, Autoimmune Diseases, Arthritis, Rheumatoid, Lupus Erythematosus, Systemic, Fibroblasts

## Abstract

Secondary lymphoid organs such as lymph nodes (LNs) are the home of peripheral tolerance mechanisms which control autoreactive T cells and prevent immune responses to self-antigen. In systemic autoimmunity, there is a clear failure of these peripheral tolerance mechanisms that leads to chronic inflammation and tissue destruction, highlighting the role for LNs as possible gatekeepers of autoimmunity. In recent years there has been a shift in research focus towards tissue sites in autoimmune diseases ranging from type 1 diabetes to rheumatoid arthritis in an effort to better characterise pathogenesis and guide diagnostic and therapeutic decisions. Although this has yielded great insight, it fails to tackle the initial break in tolerance that initiates disease progression which is most likely originating in peripheral LNs. In the majority of autoimmune diseases a preclinical phase is recognised. This is characterised by the presence of autoantibodies, which is indicative of a break in immune tolerance, and the absence of clinically apparent inflammation or tissue destruction. This review explores how our current knowledge of LNs in the preclinical and established phases of autoimmune diseases provides insight into possibly shared pathological mechanisms that drive disease progression and highlight the gaps in our knowledge that may help uncover new therapeutic avenues for intervention and prevention.

**What is already known on this topic?**

- Autoimmune diseases have a clear preclinical phase preceding pathology that suggest a break in immunological tolerance due to the presence of autoantibodies that is likely to involve secondary lymphoid organs such as lymph nodes.

**What this study adds?**

- Here, we reviewed current knowledge surrounding the involvement of secondary lymphoid organs such as lymph nodes in the pathogenesis of autoimmune disease and highlighted key areas where further research would be beneficial.
- We highlight how lymph nodes provide an additional immunological landscape to that observed in peripheral blood and sites of chronic inflammation or tissue destruction.
- We show how the loss of immunomodulatory function in lymph node fibroblasts may help steer immune cell activation and the development of autoimmune disease.
- This review conveys the importance of investigating lymph nodes in at-risk individuals as it can provide key insights into the failure of gate keeping events that leads to autoimmune disease.

**How this study might affect research, practice or policy?**

- This review article advocates for increased research efforts in investigating lymph nodes in order to advance our knowledge of the earliest stages of autoimmunity in order to guide therapeutic intervention and prevention.
- This review concludes that lymph node analysis in the context of autoimmunity needs a concerted effort to include healthy age—-matched individuals to understand the intricacies of the cellular changes that occur in pathology.

## Introduction

**Figure 1:**
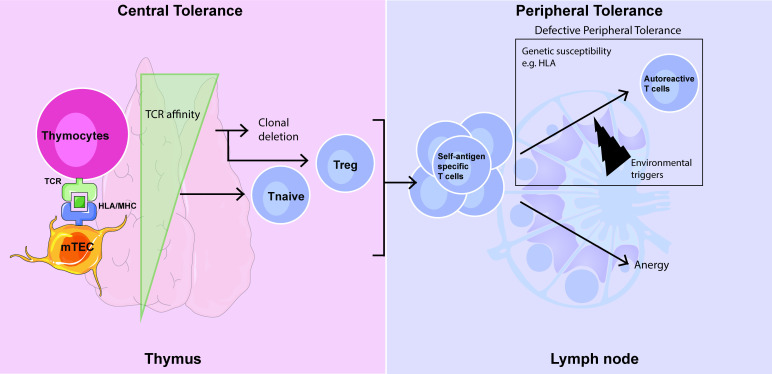
Central and peripheral tolerance. Simplified schematic overview outlining how autoreactive T cells may emerge due to malfunctioning of central and peripheral tolerance mechanisms. In central tolerance, occurring in the thymus, thymocytes are exposed to self-antigens expressed on mTECs to prevent autoreactive T cells from entering the periphery while maintaining a diverse T cell repertoire. The affinity of this interaction determines whether T cells undergo clonal deletion, become Tregs or remain naive T cells (Tnaive). Peripheral tolerance is a secondary control mechanism to suppress self-antigen-specific T cells that have escaped central tolerance and can originate from both naive and Treg of thymic origin. mTECs, medullary thymic epithelial cells; Tnaive, naive T cells; Treg, regulatory T cell; TCR, T cell receptor; HLA, human leukocyte antigen; MHC, major histocompatibility complex.

Lymph nodes (LNs) are highly organised secondary lymphoid organs that orchestrate effective adaptive immune responses to invading pathogens. They are the home of peripheral tolerance which acts as a secondary control mechanism to prevent autoreactive T cells from entering the periphery and mounting immune responses to self-antigen. A normal T cell repertoire is extremely diverse containing not only T cells capable of responding to foreign antigenic challenge but also self-reactive T cells that have escaped central tolerance in the thymus. These self-reactive T cells can be regulatory in nature or need to be controlled in the peripheral tissues. Silencing of autoreactive T cells in LNs by peripheral tolerance mechanisms is coordinated by stromal cells, dendritic cells (DCs) and regulatory T cells (Tregs).^1^ On entering the LN, T cells interact with self-antigens present on antigen-presenting cells such as DCs and stromal cells. The strength of this interaction combined with costimulatory signals determines whether T cells will become anergic, undergo clonal deletion or suppression. However, in the context of autoimmune diseases there is a clear breakdown in the body’s immune system that allows it to attack itself (figure 1).

In this review, we seek to explore the role of peripheral tolerance mechanisms in the LN as gatekeepers of autoimmunity. We will provide insight into the evidence for LN involvement as described for a number of autoimmune diseases with varying organ manifestations, clinical presentation, pathogenesis and disease onset (Box 1). We will explore how changes in these secondary lymphoid organs may help autoimmunity to develop in certain individuals which may not be apparent in peripheral blood. We will lastly expand on the gaps in knowledge that still remain and how research into LNs can help our understanding of not only pathogenesis but effective diagnosis and targeted therapeutic intervention.

Box 1 **Autoimmune diseases and their clinical manifestations**

Type 1 diabetes is a chronic autoimmune disease affecting beta cell function in islets of the pancreas leading to defective insulin production and uncontrolled glucose blood sugar levels.

Systemic lupus erythematosus is characterised by chronic systemic inflammation manifesting in multiple tissue sites including the skin, joints, kidneys and brain to varying degrees.

Rheumatoid arthritis is a chronic inflammatory autoimmune disease which clinically manifests in synovial joints and if left untreated leads to bone destruction and pannus formation.

Multiple sclerosis is a chronic disease of the central nervous system which is unpredictable in nature. Tissue damage to the myelin surrounding neurons in the brain and spinal cord leads to spasms, fatigue, depression, incontinence, sexual dysfunction and walking difficulties.

### Rationale for LN involvement in autoimmune diseases

In a number of autoimmune diseases autoantibodies have been identified in the blood of patients years before clinical manifestations of disease which indicates a clear break in peripheral tolerance before disease onset.^2^,^6^ This is termed the preclinical phase of disease and in prospective studies such autoantibody-positive individuals are considered at-risk of autoimmune disease development. This phase is characterised by the presence of autoantibodies, outlined in table 1, and in some cases heightened inflammatory markers in the blood. In type 1 diabetes (T1D), several autoantibodies against islet cell cytoplasmic proteins, glutamic acid decarboxylase (GAD-65), insulin and protein tyrosine phosphatases have been identified. The presence of a single autoantibody confers a fairly low risk of T1D development with studies showing that this risk is compounded by the emergence of additional autoantibodies and the age at which they appear.^4 7 8^

**Table 1:** Overview of autoantibodies identified in preclinical phase of autoimmune diseases

Disease	Autoantibodies present at preclinical phase	Reference
Rheumatoid arthritis	Anti-citrullinated peptide (ACPAs) antibodiesRheumatoid factor antibodiesAnti-CarP antibodies	29 12
Type 1 diabetes	Islet cell cytoplasmic proteins antibodiesGlutamic acid decarboxylase (GAD-65) antibodiesInsulin antibodiesProtein tyrosine phosphatases antibodies	^4^
Systemic lupus erythematosus	Anti-phospholipid antibodiesAnti-nuclear antibodiesAnti-Ro antibodiesAnti-La antibodiesAnti-Sm antibodiesAnti-ribonucleoprotein antibodiesAnti-double stranded DNA antibodies	^3 6^
Multiple sclerosis	Epstein-Barr virus nuclear antigen 1 (EBNA1) antibodiesGlial cell adhesion molecule antibodies	^16^

Autoantibodies identified against self-antigens in rheumatoid arthritis, type 1 diabetes, systemic lupus erythematosus and multiple sclerosis including associated references.

In a similar vein, in rheumatoid arthritis (RA) a number of autoantibodies against citrullinated proteins, carbamylated proteins and immunoglobulin G Fc region, known as ACPAs, anti-CarP and rheumatoid factor, respectively, have been identified. These are present in the preclinical phase of disease and provoke, like in T1D, increased risk of RA development which is intensified by the presence of multiple autoantibodies and environmental factors such as smoking and obesity.^29^,^13^ In the case of smoking there is additional evidence that it induces increased citrullination of peptides through expression of the peptidylarginine deaminase 2 enzyme within the lung.^14^

In other autoimmune diseases the presence of autoantibodies and their relation to risk and development is less clear cut. In multiple sclerosis (MS), the presence of autoantibodies once thought to be unique to MS have since being subcategorised into rarer demyelinating diseases of the central nervous system (CNS), such as autoantibodies against myelin oligodendrocyte glycoprotein (MOG) which distinguishes MOG-antibody associated disease. Several autoantibodies have been identified but have not been definitively shown to be specific to MS but rather indicative of a broader antibody response.^15^ Although the identification of autoantibodies has been elusive, the evidence for increased cross-reactivity of antibodies from patients with MS with Epstein-Barr viral antigens does suggest that the disease may emanate from molecular mimicry as a result of post-translational modification of self-proteins.^16 17^

Systemic lupus erythematosus (SLE) is another autoimmune disease where the autoantibody landscape is unclear. Autoantibodies against nuclear proteins, double-stranded DNA and smith (Sm) protein have all been implicated in SLE; however, like in MS they are not all solely attributed to SLE development. In SLE, the presence of doubled-stranded DNA autoantibodies have been strongly associated with lupus nephritis. These autoantibodies are thought to be pathogenic via complement activation and cross-reactivity with kidney self-antigens.^18^,^20^ The underlying mechanisms that orchestrate the resulting kidney damage needs to be investigated further. SLE autoantibodies are also known to fluctuate during disease and the presence of both anti-double-stranded DNA and anti-Sm antibodies have been associated with a higher risk of disease flare.^6 21^

These examples highlight the heterogeneity of the involvement of autoantibodies in autoimmunity and the difficulty in studying them. Considering the fact that autoantibody presence does not give rise to autoimmune disease^3 4 22^ in all individuals means there are likely secondary mechanisms to evoke pathogenicity which have yet to be fully elucidated. The growing concept of epitope spreading which confers benefit in viral immunity is thought to contribute to pathogenicity in RA^23 24^ and other autoimmune diseases and help delineate progressors in at-risk populations. Notwithstanding, the presence of autoantibodies in these autoimmune diseases does point to a causal role for peripheral LNs, as these are sites of initial (auto)antibody production. This production may be driven by a break in immunological tolerance for which the biological mechanism is currently unknown.

### Defective peripheral tolerance in the LN

**Figure 2:**
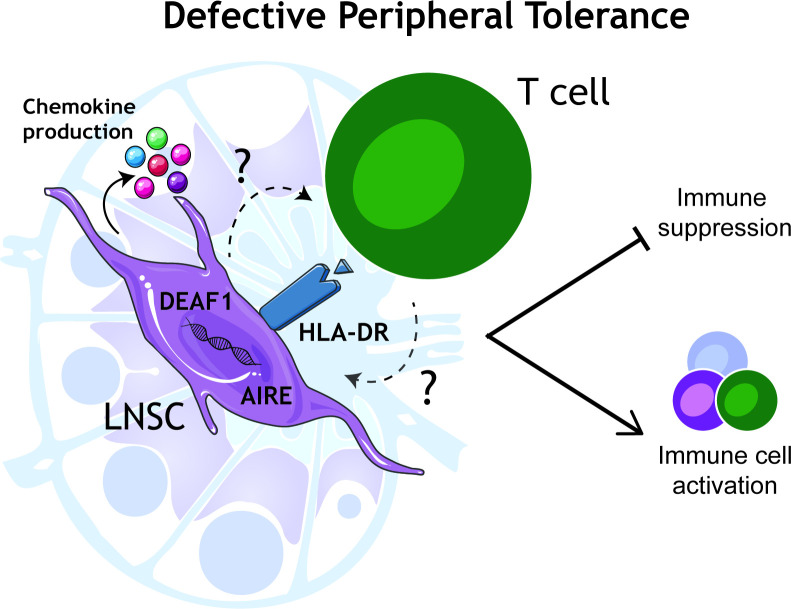
Proposed defective LNSC-mediated peripheral tolerance in autoimmune diseases. This schematic highlights the common changes in the immunomodulatory function of LNSCs that may be at play in immune cell activation that subsequently contribute to clinical manifestations in disease-specific target organs. DEAF1, deformed epidermal autoregulatory factor 1; LNSCs, lymph node stromal cells.

Deciphering the immunological changes in the LN that aid this break of peripheral tolerance and autoantibody production are critical to our understanding of autoimmune diseases. These changes are likely to provide greater insight than has been captured thus far by extensive immunophenotyping of peripheral blood. In the last decades, it is becoming increasingly apparent that the pathogenic cells driving autoimmune diseases exist in target tissue sites and probably migrate from secondary lymphoid organs. Here tissue resident cells due to a combination of genetic susceptibility and environmental factors may become predisposed to promoting an inflammatory environment (figure 2).

Antigen-presenting cells within the LN have been an area of interest in a number of autoimmune diseases as possible drivers of peripheral tolerance loss. DCs are key antigen-presenting cells that allow the body to mount an effective adaptive immune response through presentation of foreign antigen to T cells. Resident DCs are also capable of expressing peripheral tissue antigens thereby inducing tolerance and preventing autoreactivity.^25^ Interestingly CD1c+ myeloid DCs and CD304+ plasmacytoid DCs were shown to only be increased in patients with RA and not in autoantibody positive at-risk individuals.^26^ Similarly decreased follicular DC frequency alongside reduced follicles have been observed in recently diagnosed patients with T1D but not in autoantibody positive at-risk individuals.^27^ These observations imply that DCs may be more involved in the latter stages of disease, perpetuating chronic inflammation rather than initiating events. However, as not all DC subsets have been studied during the preclinical phase of disease, future studies are needed to confirm this.

Tissue-resident LN stromal cells (LNSCs) have also been of interest due to their recently discovered role in immunological tolerance.^28^ LNSCs provide structural support and help maintain LN architecture under homeostasis through the secretion of chemokines such as CCL19, CCL21^29^ and CXCL13^30^ to promote T and B cell migration, respectively, as well as providing IL-7^31^ to promote T cell survival. Murine studies have highlighted a role for LNSCs in presenting peripheral tissue antigens (PTAs)^32 33^ under homeostatic conditions. The evidence suggests that, like in the case of medullary thymic epithelial cells in the thymus,^34^ specific LNSC subsets express unique combinations of PTAs. In the last decade, mouse studies have implicated LNSCs in peripheral tolerance. LNSCs have the capacity to delete self-reactive CD8+ T cells through PTA expression and maintain regulatory CD4+ T cells under homeostasis.^35^,^39^ Investigating peripheral tolerance in humans can be very challenging; however, LN tissue studies in autoimmune diseases provide a great opportunity to learn about the disease initiating events and also explore the fundamentals of peripheral tolerance in humans. Of interest, DNA methylation analysis of LNSCs from patients with RA and at-risk individuals have highlighted alterations in gene regions associated with antigen processing and presentation when compared with healthy individuals.^40^ LNSCs express the key transcription factors autoimmune regulator^41^ and deformed epidermal autoregulatory factor 1 (DEAF1) which are both involved in PTA expression. Furthermore, LNSCs from at-risk and patients with RA have been shown to express HLA-DR and other costimulatory molecules illustrating that these cells have the machinery to express and present self-antigens.^41^ Furthermore, ACPAs isolated from patients with RA can bind proteins present in LN biopsies and LNSCs of RA patients and at-risk individuals.^41^ Together this evidence suggests a dysregulated stromal cell environment in RA LN, where altered LNSCs may express self-antigens inducing immune cell activation rather than tolerance (figure 2).

Similarly in T1D, alternatively spliced variants of DEAF1 have been identified in the pancreatic LNSCs of patients with T1D and autoantibody positive at-risk individuals.^42^ This loss of functional DEAF1 due to alternative splicing may contribute to peripheral tolerance loss in the context of T1D. In RA, although DEAF1 has been identified in LNSCs, splice variants have not been investigated. In T1D, LNSCs isolated from pancreatic LNs showed increased expression of HLA-DR in autoantibody positive at-risk individuals and patients with T1D compared with healthy controls.^43^ This HLA-DR expression was also observed to be higher in autoantibody at-risk individuals compared with established disease, which we have also observed in the inguinal LN of RA-risk individuals when compared with patients with RA (submitted O’Byrne *et al* 2024). This altered HLA-DR expression may tip the balance towards a more activated LN environment (figure 2) capable of activating rather than tolerising self-antigen specific T cells; however, this is ultimately hard to prove. Further experiments exploring co-stimulatory molecule expression combined with assessing cytokine production are required to determine the impact on surrounding immune cells.

Subsequently there has been a concerted effort to identify whether autoantigens are present in proximal LNs during autoimmunity. This has been challenging due to the specificity of autoantigens within each disease and the accessibility to the draining LN in question. In the case of T1D, murine studies provided very early evidence for LN involvement as the removal of LNs prevented onset in the non-obese diabetes mouse model^44^ and islet specific T cells were identified preceding onset.^45^

In RA, immunohistochemistry revealed the presence of peptidylarginine deiminase (PAD) enzymes and citrullinated proteins in the inguinal LN of patients with RA and autoantibody-positive individuals suggestive of autoantigen presence in the LN and a possible site of citrullination.^41^ These citrullinated antigens were also shown to be expressed in LNSCs^41^ which may help perpetuate an immune response to these modified self-antigens.

In MS, increased presence of myelin antigen-containing cells were observed in the cervical LN of patients compared with control individuals which in rhesus monkeys were also shown to express markers for antigen presentation.^46^ Additional studies have shown the presence of myelin basic protein and proteolipid protein in macrophages within the cervical LN of patients with MS and only in a minority of healthy controls.^47^ In more recent years studies exploring a wider set of neuronal antigens revealed them to be present in large medullary cells deemed macrophages morphologically with some neuronal antigen-expressing cells also expressing MHC-II and CD40 which are key markers for antigen presentation.^48^

The presence of autoantigens and autoantibodies in autoimmunity and their apparent prevalence in the LN has resulted in numerous studies focused on identifying antigen-specific T and B cells. In particular T cells are vital to mounting effective adaptive immune responses to invading foreign pathogens and driving B cell activation and ultimately immune resolution. In the context of autoimmune disease such cells are capable of auto reactivity and in turn pathogenicity.

### Antigen-specific T cells

As is the case with autoantibodies, it is apparent that the presence of T cells specific for self-antigens is not conducive to developing an autoimmune disease and are not necessarily pathogenic in the preclinical at-risk phase.

For example in T1D, self-antigen-specific T cells recognising insulin epitopes have been found in the peripheral blood of healthy individuals which only in the case of patients with T1D are also present in the pancreas.^49^ Another study identified pre-proinsulin-specific CD8+ T cells in the pancreas of healthy individuals as well as newly diagnosed T1D and autoantibody positive at-risk individuals. However these CD8+ T cells were shown to be proximal to islet regions in newly diagnosed T1D but evenly spread in healthy individuals.^50^ It is likely that a second islet-specific trigger alongside genetic predisposition permits them to become pathogenic in T1D.^50^ Furthermore it is clear that these autoreactive T cells go on to perpetuate disease as they are still abundant in the pancreas years after onset.^51^ Antigen-specific T cells have also been shown to reside in pancreatic draining LN of human organ donor material of patients with T1D.^52^ An early study by Sibley *et al* in 1985 suggests that these antigen-specific T cells likely form a reservoir in LNs which may help perpetuate disease. In this small study, four patients with T1D received islet pancreatic transplants from their HLA-matched twin or sibling, in one case, which still led to organ rejection^53^ due to islet destruction and lymphocyte infiltrate.

In RA, antigen-specific T cells against a number of self-peptides, which have undergone citrullination including tenascin C,^54^ alpha endolase,^55^ type II collagen^56^ and vimentin^57^ have been identified in blood and synovial fluid and in the case of citrullinated tenascin C in synovial tissue too.^58^ Antigen-specific T cells against citrullinated vimentin have also been shown to be CD8 positive and cytotoxic in nature helping to drive the disease.^59^ In the inguinal LN of patients with RA and autoantibody-positive at-risk individuals, antigen-specific T cells against citrullinated vimentin, alpha endolase and fibrinogen have been found, suggesting, like in T1D, that the draining LN may serve as a reservoir to perpetuate disease.^60^

Nuclear antigen-specific T cells have also been observed in the blood and enriched in the urine of patients with SLE with lupus nephritis, a common inflammatory manifestation of the disease.^61^ These antigen-specific T cells were shown to be present in healthy individuals, patients with inactive SLE and specifically enriched in patients with active SLE.^61^ In the context of LNs there is little known about the presence of antigen-specific T cells in SLE which makes it difficult to distinguish whether these T cells are perpetuators of disease or a consequence of the chronic inflammatory environment where they have been identified.

The landscape for antigen-specific T cells in autoimmunity is vital to our understanding of autoimmune disease development but is complicated to investigate with current methods. We are still yet to determine how exactly these antigen-specific T cells, present in healthy individuals, become pathogenic and whether this is common among autoimmune diseases. Although studies in the context of RA have shown changes in antigen-specific T cells on successful treatment,^60^ it remains difficult to study pathogenicity from peripheral blood sampling as it is likely that autoreactivity is induced at tissue-specific sites. Molecular mimicry has been hypothesised as the triggering event due to cross-reactivity of viral and self-antigens following viral infection,^16 62^ which in genetically susceptible individuals leads to autoimmunity. An alternative hypothesis is that damage following infection or physical injury leads to the release of self-antigens and damaged associated molecular patterns which result in autoimmunity and inflammation. Ultimately the secondary trigger is extremely challenging to prove definitively but could help to characterise at-risk individuals for early therapeutic intervention. Notwithstanding, the current evidence for antigen-specific T cells residing in the LN supports the idea of this secondary lymphoid organ as a gatekeeper of autoimmunity.

### Evidence of activated LNs in autoimmunity

#### Lymphadenopathy

**Figure 3:**
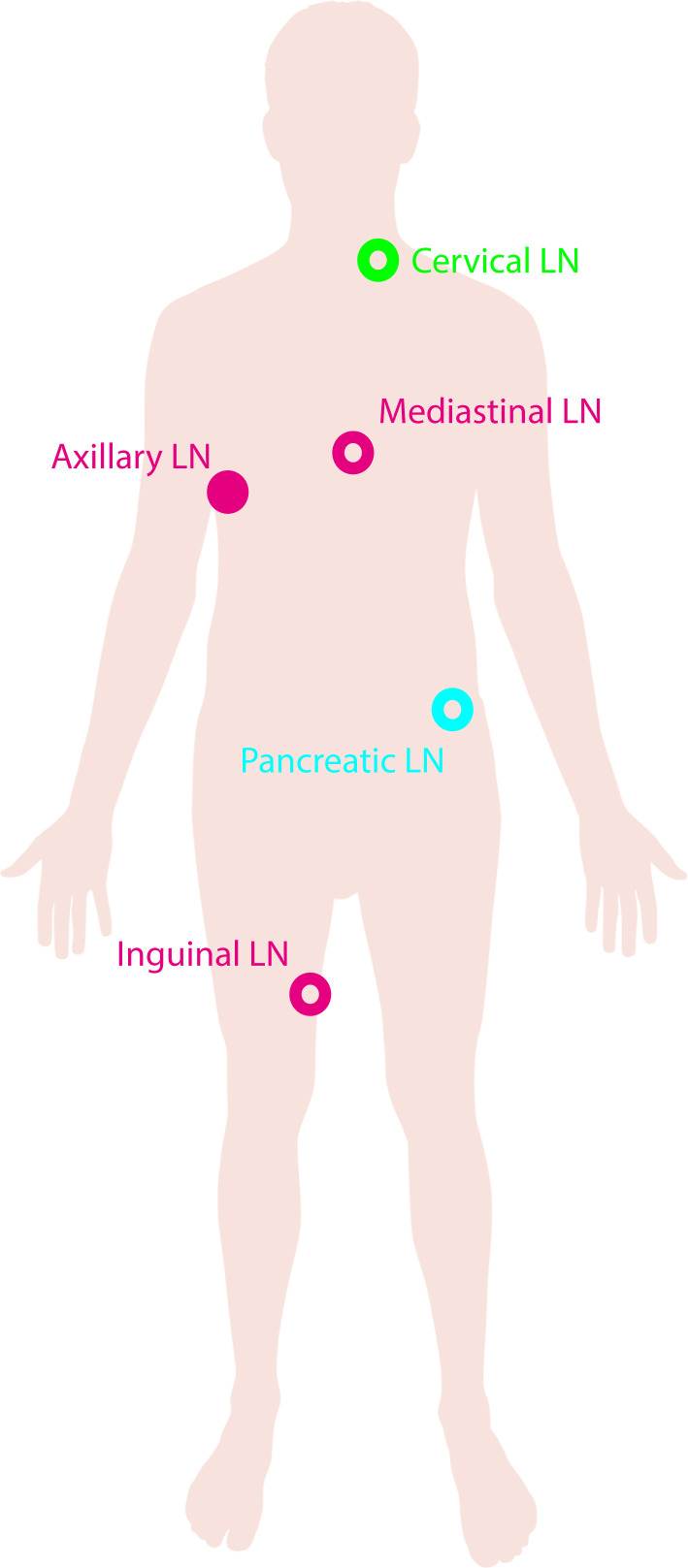
Anatomical locations of LNs studied in autoimmune diseases. Schematic showing the location of LNs investigated proximal to sites of autoimmunity. Axillary, mediastinal and inguinal LN (pink open) in rheumatoid arthritis, axillary LN (pink closed) in systemic lupus erythematosus, pancreatic LN (blue) in type 1 diabetes and cervical LN (green) in multiple sclerosis. LN, lymph node.

The presence of autoantigens and antigen-specific T cells residing in the LN indicates an altered and possibly activating LN environment in autoimmunity. To this end, lymphadenopathy, regarding the swelling of LNs, has been implicated in a number of autoimmune diseases (figure 3).

In MS there has been studies to investigate cervical LN (figure 3) size and how it may correlate with clinical parameters such as disease progression and therapeutic response. Cervical LN enlargement, assessed by ultrasound, has been observed in patients with MS compared with age-matched controls which did not correlate with the ongoing treatment.^63^ In contrast, a reduction in axial cervical LN diameter, measured by MRI, has also been associated with disease progression with larger diameters correlating with shorter disease duration.^64^

In SLE, generalised lymphadenopathy is more commonly observed and associated with higher disease burden and the presence of anti- double stranded DNA (dsDNA) antibodies but not associated with renal or CNS involvement.^65^ The generalised lymphadenopathy may be reflective of the multiorgan involvement observed in SLE compared with diseases such as RA and T1D which have a more localised organ involvement. In a minority of cases more localised lymphadenopathy is observed in younger patients with more active disease.^65^ Lymphadenopathy is not currently included in diagnostic criteria for SLE, due to its lack of specificity as it is also observed with similar histological presentation in rarer diseases such as Castleman Disease and Kikuchi-Fujimoto disease.^66^

In RA, lymphadenopathy has been observed in up to 82% of patients,^67 68^ which is localised to LNs proximal to active arthritis joints and tend to be larger than in SLE.^67^ Lymphadenopathy in the mediastinal LN (figure 3) has been associated with frequency of RA-related interstitial lung disease^69^ which further supports the idea that the enlargement observed in autoimmune diseases is dependent on specific tissue/organ involvement. In like manner, lymphadenopathy of axillary LNs (figure 3) has also been associated with local joint involvement including tender and swollen joint counts.^70^

In Sjogren’s syndrome (SS), lymphadenopathy is known to be localised to the cervical LN and possibly associated with salivary gland swelling and the presence of some autoantibodies.^71^ However very little is currently known about the cellular components of the cervical LN in SS which definitely warrants further investigation.

The extensive presence of lymphadenopathy in autoimmune diseases is evident; however, in the majority of cases, possibly excluding MS, this presence is rarely specific enough to be considered a diagnostic factor. The strong presence of lymphadenopathy in established disease is likely reflective of chronic inflammation. Evidence to date suggests that it is important to rule out the lymphadenopathy as a consequence of malignancy, particularly in the case of SS, infection or as a major defining feature of rarer diseases, such as Castleman disease and IgG4-related diseases, where aetiology and autoimmune components are even more elusive. A clear focus on the specific LNs proximal to inflammatory sites that characterise each of these diseases provides a more accurate picture. This, in turn, explains why in SLE, where multiple tissue sites are involved, there is more generalised lymphadenopathy. These findings provide compelling evidence for further investigation into LN changes and suggest an activated tissue site with increased immune cell infiltration.

#### Germinal centres in the LN

Due to the presence of autoantibodies, investigation into germinal centres has been a major focus of LN studies. Increased frequency of CD19+ B cells has been observed in the inguinal LN^72^ of biological naive patients with RA and autoantibody positive at-risk individuals compared with age-matched healthy controls. This CD19+ B cell frequency has been correlated with T follicular helper (Tfh) cell frequency.^73^ Increased frequency of CXCR5+CD4+ and CD8+ T cells were also observed in RA and autoantibody positive at-risk individuals compared with healthy controls.^73^ These data are indicative of active germinal centres (GCs) early in disease pathogenesis when immune tolerance is broken.

This may be in contrast to the evidence currently available investigating GCs in the pancreatic LN (figure 3) of patients with T1D. Pancreatic LN of patients with recent-onset T1D have altered architecture with a loss of GCs and follicular DCs.^27^ More diffused structures and poor localisation of T and B cell compartments were observed in both patients with recent-onset T1D and autoantibody positive at-risk individuals but not in non-diabetic controls or patients with T1D with a longstanding disease duration. This suggests an important event occurring at onset which contributes to disease development.^27^ Of note, the total frequency of B cell follicles did not differ nor did it stratify based on age.^27^ When stratified for immune cell infiltration to the pancreas, known as insulitis, there were no differences observed based on disease duration. This may suggest a causative role for immune cells from the LN infiltrating the pancreas; however, this cannot be definitely proven in the human context. Studies exploring the B cell compartment within the LN show an enrichment of class-switched B cells compared with the blood but failed to show this enrichment compared with the LN of healthy individuals.^74^

There is very little known about the cellular composition of LNs in MS. A preliminary study in MS investigated GC differences in the cervical LN of newly diagnosed patients with MS and healthy controls using fine-needle aspirates. This study combined single-cell RNA and cellular indexing of transcriptome and epitope sequencing to allow for both cell surface marker and transcriptomic expression and showed a loss in the proportion of GC B cells and Tfh cells.^75^ This may indicate defective GC formation like observed in T1D and not RA or increased migration to other tissue sites; however, this needs to be validated in further studies using larger patient cohorts with age-matched healthy controls.

The differences as to whether GCs are enriched or not may reflect the level of involvement of autoantibodies in disease progression and their pathogenicity. Furthermore, differences between LN studies may be explained by the location of the LN sampled and its proximity to the target tissue site involved. In the context of RA, multiple joints can be involved where autoantibody production may help drive systemic disease progression, whereas there are not multiple sites of tissue inflammation in T1D and MS. It would be interesting to explore in other systemic autoimmune diseases such as SLE whether this holds true. It is intriguing to postulate whether this loss in GCs observed in LNs draining the target organ relates directly to the GC formation at sites of tissue inflammation in the affected organ. Further exploration into the state of GC activity in autoantibody-positive at-risk individuals may help delineate whether it is a phenomenon more commonly associated with the initial break in immune tolerance rather than perpetuation to clinical disease. Overall, LN studies focusing on the phenotype and function of B cells in autoimmunity are lacking. Exploring B cell changes within lymphoid organs, in particular within the bone marrow, may highlight even earlier changes contributing to the loss of immunological tolerance observed in these diseases.

#### Additional LN activation signals

Other immune cell changes in the LN also point towards a dysfunctional LN that may contribute to defective peripheral tolerance. Reduced cytokine production has been observed in-vitro in CD4+ and CD8+ T cells isolated from inguinal LN biopsies of RA risk and RA patients compared with age-matched healthy controls with a skewing towards a Th1 phenotype shown by increased CXCR3 expression.^76 77^ In T1D LNs, no changes were observed in helper T cell subsets^74^ which may be reflected by the CD8-mediated nature of T1D.

The story for Tregs in the LN of T1D remains conflicting with no significant changes in the frequency of total Tregs in the inguinal LN of patients with recent-onset T1D compared with healthy controls.^74^ Nevertheless there is evidence for a loss in functionality shown by a slight but statistically significant reduction in CD45RA-FOXP3+ Tregs considered activated Tregs^74^ and FOXP3 demethylation^78^ which was suggested to promote a Th17 proinflammatory environment. A recent study showed a slight enrichment in the frequency of CXCR5+FOXP3+, considered T follicular regulatory T cells (Tfr), compared with healthy controls.^79^ However the lack of age-matching in this study in combination with the known increase in Tfr in aged mice makes it difficult to attribute this change specifically to T1D development. Although the frequency of IL-10 producing CD4+ T cells have been shown to be reduced in LNs of at-risk and patients with RA,^76 77^ the frequency of FOXP3+CD4+ Tregs have yet to be determined and CD8+Tregs remain unchanged.^76^ In MS, the role of Tregs in the LN have not been extensively explored but do not seem to differ between healthy controls and patients.^75^

As is well known in the human Treg field, studies of these populations in LN are hampered by the challenge to find a definitive phenotypic Treg marker that is as robust as FOXP3 in the mouse. As a result the picture on Treg frequencies in autoimmune diseases remains muddled due to the range of different markers used to define the Treg subset which makes comparisons between studies challenging.

### What more can we learn from the LN?

The increasingly apparent differences between the immune landscape of peripheral blood compared with the LN^72 74 77^ highlights how the LN may provide a clearer outlook in terms of therapeutic responses. Although therapeutic studies are scarce, LN biopsy analysis has been shown to uncover incomplete B cell depletion in patients with RA treated with rituximab, an observation not reflected in the peripheral blood.^80^ Future therapeutic studies involving LN biopsies may aid our understanding of the variable therapeutic responses observed in many autoimmune diseases. They can also provide clues as to the role of antidrug antibodies in impeding therapeutic efficacy which are likely produced by GCs of LNs. Given the observable changes that have been outlined in this review, the LNs may also be a valuable target for biomarker discovery in at-risk populations to accurately predict disease progressors. LNs can also provide an environment for targeted treatment. Recent murine studies employing intra-LN injection of tolerogenic micro particles were able to induce antigen-specific tolerance.^81^ This concept may be applicable in future treatment of autoimmune diseases following further research. The recently observed efficacy of bispecific antibodies, in refractory RA, which are able to bind both B and T cells^82^ could indeed be more effective if targeted towards the LN where we know both lymphocyte populations are present and likely inducing pathogenicity.

As outlined above, there is clear evidence that exploring the LN can be insightful and beneficial to our understanding of autoimmune diseases. It is important to highlight the difficulties in studying human LNs in autoimmunity, though it is common practice in the oncology field. Sampling of human LNs is challenging as they can only be obtained via surgery, autopsy and needle biopsy which in the case of the latter does not always result in sufficient cell yield for downstream functional analysis. Furthermore, the exact locations available for sampling can be limited which can have disease dependent compounding effects. For example, the tissue organ draining LNs in the context of RA compared with T1D differ significantly in their accessibility. In T1D, LN studies assess the pancreatic draining LN closest to the inflammatory site compared with RA where the closest LN differs significantly in individuals based on their affected joints, making subsequent comparisons challenging. We know in RA that there are systemic changes occurring irrespective of arthritis location^72^ likely reflecting systemic autoimmunity. It would be interesting to investigate distal LNs in organ-specific autoimmunity like T1D to see if LN changes are location dependent. In the case of multiple organ involvement, like in SLE, it is important to take into account systemic changes in the LN as well as those that may be specific to certain clinical manifestations. Despite the knowledge that LN sampling is well tolerated by volunteers within both patients and at-risk individuals,^83^ the large investment into infrastructure required also makes LN studies challenging. Nevertheless, there have been a few recent studies exploring the immunological landscape of healthy human LNs under homeostatic conditions. These studies used either fine-needle aspirates^84 85^ or whole LN sampling^86 87^ from cadavers to explore the immune cell compartments of LNs from various anatomical locations. Notwithstanding these important contributions, studies of healthy human LNs remain sparse, given the ethical implications, which can complicate analysis of immunological LN changes in disease states. Spatial technologies to map the immune cell compartments have made major inroads in oncology.^88^ Although in their infancy in the context of autoimmune diseases these technologies will provide much needed clarity on LN biology in autoimmunity. Despite these challenges, continued multidisciplinary efforts, including the application of novel spatial technologies, to determine the contribution of LNs as gatekeepers of autoimmunity will be paramount to unravelling the pathogenesis of these complicated multifactorial autoimmune diseases.
